# Dothinenteritis—Dr. Bretonneau's New Theory of Fever

**Published:** 1826-07

**Authors:** 


### Dothinenteritis—New Theory of Fever.

Well might Cicero say—"opinionum commenta delet dies." What part of the body will escape the honour, or rather the opprobrium, of being the seat of fever? We shall see in time! Certain glandular bodies, which have been described by Haller and others, but particularly by Peyer, occupying the small intestines: more numerous in the neighbourhood of the ileo-cæcal valve, and spreading upwards, with gradual diminution of size to the middle of the jejunum, present themselves in the form of bands or stripes (which have been looked upon by some as pathological alterations) varying from six to ten lines, sometimes to that of several inches, in length. These are now pitched upon by Dr. Bretonneau, a distinguished physician of Tours, as the seat of a large class of fevers—such as the febris putrida genuina—the synochus putrida—the "fièvre muqueuse adynamique" of Pinel—the "gastro-enterite" of Broussais—the typhus mitior et gravior, which has ravaged Ireland, for instance, of late years—and which is never absent from the hospitals of Paris. Some of our readers will be ready to cry out—read no more—listen not to such eccentricities. But it is the part of wisdom to listen and endeavour to learn, even from the most absurd speculations. The attempts to fix the seat, and establish a theory of fever, have invariably enlarged the boundaries of our knowledge respecting that important scourge of the human race. These investigations, if they have not determined the causes, have elucidated the effects or consequences of fever, which are often more fatal than the fever itself. In failing to establish the truth of their peculiar hypotheses, they have, at least exposed the errors of others, which is no mean advantage—but they have done a great deal more. They have opened out new views of the various conditions which both cause and result from, fever, and have consequently enlightened the dark paths of practice and drawn our attention to the dangers which were before concealed from our sight. Of all parts of the body, the internal surface of the intestinal canal has most escaped the examination of pathologists, especially in this country—and yet, it is the very part which most frequently shews the ravages of the disease. It is in vain to deny this assertion. Till within the last few years, the mucous membrane in question was not sufficiently attended to in this or in any other country—and, therefore, we have no right to shut our eyes against delineations of its pathology, however fanciful may be the deductions drawn from its morbid conditions in fever. Let us listen to facts, and draw our own conclusions. It is hardly necessary to remark that the meaning of the term here employed by Dr. Bretonneau is "*pustular* enteritis," from δοθιην, pustula.

Our author has not been able to make any dissection prior to the *fifth* day of the disease, and consequently his pathological descriptions commence from that epoch. The glands of Peyer (above-mentioned) particularly in the neighbourhood of the ileo-cæcal valve, are, at this period, much swollen, their edges rising in relief above the mucous membrane—their surface rather unequal, and their length and breadth increased. The glands of Brunner (which are of a different order—are about the size of millet-seed—and scattered indiscriminately throughout the whole line of stomach and intestines, great and small) begin, at this period, to become prominent, and it is sometimes possible to distinguish the orifices of their mucous crypts. At this time, the mesenteric glands begin to take on a slight rosy tint. *6th day*, The tumefaction of Peyer's glands is increased—and, on holding a portion of intestine between the eye and the light, with the peritoneal coat next the observer, the follicular band, or stripe occupied by the said glands, will be easily distinguished by its greater opacity—and by its greater lacerability. Sometimes, but only rarely, these bands will be seen encircled by an areola of inflammation at this period. At this time, the glands of Brunner (naturally like millet-seed) are still more prominent, so that the mucous membrane now appears as if covered with a close pustular eruption. The mesenteric glands are still farther augmented in size, and heightened in colour. Between the 7th and 9th days, the glands go on increasing in size, and the inflammation reaches the crypts, which hitherto were unaffected.

*9th day*. The glands of Peyer, now more rounded out, present salient and undulating borders—are red, fungous, softened, unequal, but as yet shewing no trace of erosion. The same may be said of the glands of Brunner. The mesenteric glands are now enlarged to the size of a pigeon's egg, in general, and they are still deeper in colour and softer in substance. *10th day*, One of two things now takes place—either the phlegmatia turns (which is the most common case) towards resolution, or it proceeds on its course to run through the remainder of its stadia,

In the *first* case, the glands begin to diminish in size on the 10th day, and continue to diminish till the 14th—and, by the end of the third septenary period, there is scarcely any thing to be seen different from the healthy condition—excepting in the mesenteric glands, which preserve, for some weeks, a size above the common medium. In the *second* case, or where the phlegmasia runs through the whole of its periods, the glands of Peyer, on the 10th day, present a rugous surface, and the tissue of the follicular mass is red, thickened, and, as it were, fleshy. Some of these glands, however, as well as those of Brunner, change into a state of resolution, while the others go on their course. The mesenteric glands begin to diminish in volume, but continue of a considerably intense colour. *11th and 12th days*, The tumefaction of Peyer's glands is still farther augmented—the inflamed parts are elevated in the form of conical fungosities, shewing, on their summits, marks of slight erosion. On cutting into one of these tubercles, we find it composed of a reddish tissue, in which it is difficult to discover any trace of organization. The mesenteric glands are still farther diminished in size. *13th and 14th days*. The tumefaction is still more considerable—the summits of the tubercles excoriated and tinged with bile, which, at this period, seems to be abundantly secreted. A portion of each tubercle now appears to be in a completely disorganized state. *15th day*. About this time the dead portion of tubercle turns off, leaving a large ulcer, in the middle of which is a dead slough still adherent at the base. An inflamed areola sometimes encircles the ulcerated gland. *16th day*, The core now becomes completely detached, or is removed by the slightest effort, leaving a deep excavation, with ragged, raised, and everted edges—the bottom of the ulcer resting on the muscular, or even on the peritoneal coat, which is sometimes perforated in this manner. Frequently five or six of these ulcerations may be seen in one patch or stripe of Peyer's glands, giving it a fungous and irregular aspect. Here and there may also be seen little isolated ulcerations, having their seat in the crypts of Brunner's glands. At this time, the mesenteric glands are observed to be softened in general, and resolved into a kind of boullie. Often at the bottom of these ulcers may be seen the meseraic vessels quite bare, and which, by bursting, during life, give rise to those discharges of blood which are attributed to extinction of vitality in the internal organs.

But it is not to be concluded that all the pustules which do not take the favourable turn on the tenth day, run on thus to ulceration and sloughing. It is only a small proportion, in fact, which take this course. The majority either disappear in a few days—or, remaining stationary for some time, slowly disperse, without suppuration.

*17th and 18th days*. The edges of the ulcers subside, and become less irregular—the ulcer diminishes in depth—its bottom becomes covered with the *debris* of the tissues mixed with bile and mucus. The tumefaction surrounding each solution of continuity now begins to disappear. *19th, 20th and 21st days*. The glands of Peyer begin to resume their natural size, though still of red colour. The ulcerations commence the healing process—and the appearances of redness and inflammation which surrounded them, have vanished. *25th day.* The glands of Peyer and Brunner have now assumed their natural volume, and are only distinguished by a rosy tint, and by the recent cicatrizations, or by some straggling ulcers not yet healed. *30th day,* By this time the cicatrices are generally firm; yet some ulcerations remain even till this period—especially about the lower portion of ileum. *40th day.* It is very rare, but it sometimes happens, that the ulcerations are not entirely healed even at this remote period.

Such are the pathological conditions which M. Bretonneau has been a number of years in ascertaining, and which his numerous pupils and friends have had the opportunity of witnessing and verifying in Tours, Paris, and in the French armies.

We must leave the confirmation or refutation of these statements to the experience of future pathologists; but shall introduce one or two cases by way of illustration.

*Case* 1. Louisa C. aged 19 years, entered the Hospital on the 21st May, 1824; had lately been exposed, at the period of menstruation, to cold, which suppressed the discharge. From this time she had periodical head-ache, pain in the loins, vomitings, dry cough. On the 16th May, she had cold chills, and general mal-aise, and next day intense fever. This state continued till the date of her entrance into Hospital, as above. She now presented the following phenomena—vacillating pulse, which was very quick—tongue moist and red at the point—respiration interrupted—sharp, dry cough—pains in both sides of the chest—head-ache intense. *Bled to* 16 *ounces.* Blood not inflamed. 18th, Stupor—decubitus supinatus—cheeks vivid red—breathing less frequent than yesterday—pulse depressed, quick, irregular—tongue red at the point, white and cheesy towards the root—skin very red yet moist. *Diluent drinks.* 19th, Same symptoms, but the stupor is more profound. Twelve leeches to the fundament. Hitherto the alvine evacuations were natural, and there was no thirst, no delirium. 20th, The cerebral symptoms predominant—stupor and apathy extreme. The diagnosis was now *acute hydrocephalus.* Twelve leeches to the neck. 21st. Dilatation of the pupils —tongue vivid red—stupor profound. Died in the course of the day.

*Dissection.* Neither in the head nor in the chest could the least mark of morbid structure be perceived, though the greatest care was taken in the dissection. There was no inflammation in the stomach. About eight feet from the termination of the ileum the eruption which we have described, commenced, and was of a very confluent character. The glands of Peyer and Brunner were swollen, but presented no trace of erosion. The mucous membrane between the glands was perfectly colourless. The eruption was very visible in the cæcum, and in the ascending and transverse arches of the colon.

*Remarks.* This case is valuable in another point of view besides that of the eruption abovementioned. We see that the affection of the head was at one time such that the disease was pronounced *acute hydrocephalus*; yet no trace of disease was discoverable on dissection!

*Case 2.* Gerard, a soldier in the 9th dragoons, aged 24 years, entered the Hospital of Tours, on the 7th February, 1824. The account received with him was, that, ten days previously, he had experienced alternate chills and flushings, head-ache, moderate fever, diarrhœa. 8th (day after reception) and 12th day of the disease—decubitus supinatus—air of drunkenness—prostration—stupid look—tongue red at the point and furred elsewhere—teeth dry and rather encrusted—pulse small, undulating—subsultus tendinum—deafness—delirium—stools frequent—thirst intense—abdomen soft. *Lavements—rice water.* *13th day of the disease.* The above symptoms are all exasperated. *Same treatment.* *14th day,* Sensible amelioration. *16th day.* All the bad symptoms again in force. Pupils contracted. Stupor and prostration of strength extreme. *18th day.* Death took place this day.

*Dissection.* The membranes of the brain were transparent and healthy, and no serous or other extravasation obtained in any part of the head. The cerebral mass was perfectly healthy. Nothing remarkable in the thorax. The mucous membrane of the stomach presented no particular alteration. Throughout the whole ileum, the follicular apparatus was severely affected. In some places the ulcerations nearly extended to the peritoneal tunic. The mucous membrane was sound in the interstices between the inflamed follicular stripes.

Numerous other cases are detailed; but we do not deem it necessary to introduce any more. We think the subject of this paper is highly deserving the attention of our readers.

---

